# Pancreatic Cancer Disrupts Circadian Patterns of Gene Expression in Cardiorespiratory Muscles

**DOI:** 10.1002/jcsm.70383

**Published:** 2026-09-23

**Authors:** Jeremy B. Ducharme, Martin M. Schonk, Miguel A. Gutierrez‐Monreal, Leonardo F. Ferreira, Karyn A. Esser, Andrew R. Judge, Sarah M. Judge

**Affiliations:** ^1^ Department of Physical Therapy University of Florida Gainesville Florida USA; ^2^ Myology Institute University of Florida Gainesville Florida USA; ^3^ University of Florida Health Cancer Institute Gainesville Florida USA; ^4^ Department of Physiology and Aging University of Florida Gainesville Florida USA; ^5^ Department of Orthopedic Surgery Duke University School of Medicine Durham North Carolina USA

**Keywords:** cancer cachexia, circadian rhythm, circadian transcriptome, diaphragm, heart, muscle atrophy

## Abstract

**Background:**

Cancer cachexia, a debilitating syndrome characterized by muscle wasting, significantly impacts survival in gastrointestinal cancers like pancreatic cancer. Emerging evidence suggests a link between cancer cachexia and disrupted circadian rhythms in peripheral tissues, including locomotor muscles. However, circadian dysregulation in cardiorespiratory muscles—whose functional decline are suspected to contribute to increased morbidity and mortality in patients experiencing cachexia—remains largely unexplored.

**Methods:**

Herein, we investigated circadian gene expression patterns in cardiorespiratory muscles during cachexia using the orthotopic KPC pancreatic cancer model. To do this, circadian transcriptomes were generated from diaphragm and heart tissues collected from Sham and KPC mice every 4 h over 24 h, beginning on Day 12 postinoculation, which, based on our prior work, reflects the onset of cachexia in this model. Rhythmically expressed genes (REGs) (*P*_*c*_ < 0.01) were identified using the LR_rhythmicity R package, which evaluates the goodness‐of‐fit (R^2^) to a 24‐h sinusoidal model of transcript oscillations. Differences in circadian patterns, including changes in amplitude, phase and basal expression, were assessed using the LR_diff R package with *p* < 0.05.

**Results:**

We found that ~60% of rhythmic genes lost their circadian rhythmicity in both tissues, with distinct shifts in gene networks. Diaphragm disruptions centred on repression in basal expression and/or amplitude of core clock components and rest‐phase‐dependent disruptions to gene networks governing lipid and oxidative programs of metabolism and proteostasis, which were linked to an upregulation and gain of rhythmicity in inflammatory networks that peaked during the rest phase. Circadian disruptions in the heart involved loss of rhythmicity in gene networks governing cardiac function, including beta‐adrenergic and cAMP signalling, cellular responses to insulin and neurogenesis, with a similar, but more limited upregulation of inflammatory networks.

**Conclusions:**

These findings demonstrate that pancreatic cancer cachexia is associated with widespread circadian dysregulation in cardiorespiratory muscles, potentially contributing to both muscle wasting and functional decline.

## 1 Introduction

Cancer is well‐established to have profound negative effects on the body. These effects may include pain, nausea, constipation, depression and fatigue. When cancer causes a loss of muscle mass and body weight, with or without accompanying fat loss, this condition is known as cachexia, which affects up to 80% of patients with cancer [1]. Cachexia is particularly prevalent in certain cancers, including pancreatic cancer where ~70% of patients exhibit cachexia at the time of diagnosis [1]. Due to skeletal muscle loss, cachexia may impair essential functions such as locomotion, postural control, and respiration, leading to diminished physical function, reduced quality of life, decreased tolerance to cancer treatments and increased surgical complications [2]. Consequently, cachexia reduces survival time in cancer patients and may account for up to 30% of all cancer‐related deaths [2]. Unfortunately, due to an inadequate understanding of the mechanisms driving cancer cachexia, effective therapeutics to treat this pathology are not currently available, except in Japan where Anamorelin is approved for improving appetite [3].

Broadly, cancer cachexia results from chronic inflammation, dysmetabolism and anorexia, with the most advanced emerging therapies targeting central feeding regulation to improve appetite [4]. Although critical, additional therapies targeting inflammation and dysmetabolism are likely needed, highlighting the importance of understanding how these processes are regulated in cachexia. The circadian clock, present in nearly every mammalian cell, is a key regulator of tissue homeostasis, driving time‐of‐day‐dependent fluctuations in metabolism and immune function through transcription factors and autoregulatory feedback loops that generate 24‐h rhythmic gene expression [5, 6]. Consistent with this, clock dysfunction is sufficient to cause inflammation [5] and dysmetabolism, including impaired glucose homeostasis and altered lipid metabolism [6]—all key features of cancer cachexia [7, 8]. Despite these connections, the role of clock disruption in cancer cachexia is not well understood. In cancer research, circadian clock disruption has been observed in breast, lung, liver, colon and pancreatic tumours, where it is linked to metabolic rewiring and increased growth [9]. However, the impact of cancer on circadian clocks in tissues peripheral to the tumour is less well explored.

The few studies investigating peripheral clocks in tumour‐bearing hosts have mainly focused on the liver, a central metabolic tissue whose dysfunction is linked to cachexia in mice and people with cancer [10, 11]. Masri, Hojo and colleagues demonstrated disruptions to the liver clock in preclinical breast [10] and lung [11] cancer models, which contributed to increased glucose production, identifying a potential metabolic consequence of circadian disruption linked to cachexia. In addition, recent work from our lab in the mouse *tibialis anterior*, a peripheral locomotor muscle, revealed disruptions in circadian patterns of gene expression regulating glucose, lipid and oxidative metabolism in response to pancreatic cancer, which was rescued in skeletal muscles spared from cancer‐induced muscle loss through deletion of the Forkhead Box P1 (FoxP1) transcription factor [12]. Together, these studies suggest that circadian disruption in peripheral tissues of tumour‐bearing hosts may contribute to the aetiology of cachexia. However, the scope of circadian disruption in cancer cachexia is far from understood. In this regard, in addition to locomotor muscles, vital cardiorespiratory muscles such as the diaphragm and heart also undergo wasting and dysfunction in response to cancer [7, 13, 14], which is thought to be a key contributor to cachexia‐associated morbidity and mortality [15]. We therefore hypothesized that circadian programs in these tissues that uniquely govern cardiorespiratory muscle function would also be disrupted, highlighting potential avenues for therapeutic intervention.

## 2 Methods

### 2.1 Cancer Cachexia Model

Murine pancreatic KPC FC1245 cells, which derive from the tumour of a KPC (LSL‐Kras^G12D/+^; LSL‐Trp53^R172H/+^; Pdx‐1‐Cre) mouse, were gifted by Dr. David Tuveson (Cold Spring Harbour Laboratory, Cold Spring Harbour, NY). Cells were cultured in growth medium (Dulbecco's Modified Eagle's Medium with 10% foetal bovine serum and 1% penicillin/streptomycin) in a humidified chamber (37°C and 5% CO_2_).

All experiments were conducted at the University of Florida and approved by the University of Florida Institutional Animal Care and Use Committee (IACUC). Mice were housed under standard conditions with ad libitum access to food and water in a temperature‐ and humidity‐controlled facility on a 12‐h light/dark cycle. To induce cancer cachexia, the pancreas of 39‐ to 55‐week‐old male C57BL/6 mice was surgically exposed and orthotopically injected with 2.5 × 10^5^ KPC cells in 50 μL of sterile saline (KPC) or with 50 μL of sterile saline (Sham) [12]. Mice were euthanized and tissues harvested 12 days after inoculation, a time point that we have shown in two independent time course studies that correspond to the onset of cachexia in this model. In both studies, cachexia onset was defined as the time in which concurrent loss of body mass and peripheral skeletal muscle mass is first observed [16, 17], occurring several days prior to reaching IACUC‐mandated humane endpoint when mice are severely cachectic.

### 2.2 Mice and Circadian Collection

Diaphragm and heart muscles were part of a larger circadian study in which *tibialis anterior* muscles from skeletal muscle‐specific FoxP1 KO mice and WT controls (FoxP1fl/fl) on a C57BL/6 background were harvested and analysed [12]. For the current study, we expanded our investigations to determine how cachexia influences the circadian transcriptomes within diaphragm and heart muscles harvested from these mice, focusing on WT mice only. We have recently demonstrated that these mice develop significant cachexia, including body, muscle, heart and diaphragm wasting in response to KPC tumours when harvested on Day 14 postinoculation [18], with the magnitude of cachexia comparable to that induced in wild‐type C57BL/6 KPC mice at this same time point [17].

Mice were entrained to a 12‐h light/dark cycle (lights on at 6:00 AM), then placed into constant darkness starting at the transition from dark to light (circadian time 0: CT0/6:00 AM in our facility) in light‐tight circadian cabinets (Actimetrics, Wilmette, IL; RRID:SCR_025083). Tissue collections began 18 h later (CT18) on Day 12 postinoculation, a time point previously shown to correspond to the onset of cachexia in this model [16, 17] and continued every 4 h for 24 h, resulting in six time points (CT18, 22, 26, 30, 34 and 38; *N* = 2/group/time point). To maintain standardized circadian conditions [12], all tissues in the larger study (*N* = 8/time point) were collected rapidly (< 1 h) in constant darkness, precluding measurement of body and tissue weights at collection; therefore, tibialis anterior, gastrocnemius and tumour masses were obtained from snap‐frozen tissues to provide phenotypic context for cachexia in this cohort. Circadian times in this study correspond to the following times of day: CT18 = 12:00 AM (middle of active phase), CT22 = 4:00 AM, CT26 = 8:00 AM, CT30 = 12:00 PM (middle of rest phase), CT34 = 4:00 PM and CT38 = 8:00 PM. Note that time of day associated with each CT collection point will be specific to local animal facility light/dark schedules during entrainment.

### 2.3 Circadian RNA Isolation and Sequencing

Total RNA was isolated from diaphragms and hearts at each collection point following homogenization in TRIzol, extraction with phenol/chloroform and treatment with DNAse, as previously described [8]. RNA integrity was assessed using a Bioanalyzer 2100 system (Agilent Technologies, Santa Clara, CA). Bulk RNA‐sequencing was performed by Novogene Corporation (Sacramento, CA). Briefly, 1 μg of RNA per sample was used to generate libraries, which were sequenced on Novogene's Illumina NovaSeq 6000 (2 × 150 bp) to a depth of at least 40 M reads per sample. Paired‐end reads were aligned to the *Mus musculus* genome (mm39) using the STAR software (v2.5) and annotated with HTseq‐counts (v0.6.1). Differentially expressed genes (DEGs) during the active phase (CT18, CT22, CT38; *N* = 6/group) and rest phase (CT26, CT30, CT34; *N* = 6/group) were identified separately in the diaphragm and heart using DESeq2 (v1.20.0) with a Benjamini–Hochberg adjusted *p*‐value (*q*‐value) < 0.05. Functional enrichment analyses were performed using DAVID (v2021) to identify enriched Gene Ontology biological processes, KEGG and Reactome pathways (EASE score *p* < 0.05). FPKM counts for rhythmicity analysis are provided in File S1.

### 2.4 KPC RNA‐Seq Time Course Dataset

RNA‐seq data collected from diaphragm muscles harvested 2–4 h after lights on from Sham mice and mice with orthotopic KPC tumours at various stages throughout the cachexia trajectory were extracted from GEO:GSE271521 [17].

### 2.5 Statistical Analysis

Circadian transcriptomic analyses model gene expression as continuous 24‐h functions rather than relying solely on replication at individual time points (Reference S1) In this study, diaphragm and heart tissues were collected at six evenly spaced intervals across 24 h (*N* = 2/time point), yielding 12 independent RNA‐seq samples per condition (Sham and KPC). From our bulk RNA‐seq data, rhythmically expressed genes (REGs) in each muscle and condition were identified using the LR_rhythmicity R package, which evaluates the goodness‐of‐fit (R^2^) to a 24‐h sinusoidal model of transcript oscillations (Reference S1). Genes with *P*_*c*_ < 0.01 were classified as rhythmic, while genes with *P*_*c*_ > 0.10 were considered nonrhythmic to avoid misclassification of borderline transcripts (Reference S2). Differential circadian patterns, including changes in amplitude, phase, and basal expression, were assessed using the LR_diff R package with *p* < 0.05 (Reference S1). This experimental design and analytical approach adhere to established standards for genome‐scale circadian transcriptomics (Reference S3), and CircaPower estimated sufficient power (97.1%) to detect circadian rhythmicity under the current study design (Reference S4). All other data analyses were conducted in GraphPad Prism (v10.3.2). Differences in clock gene expression across cachexia stages were assessed using one‐way ANOVA with Tukey HSD post hoc tests. *p* < 0.05 was considered statistically significant.

## 3 Results

### 3.1 The Circadian Transcriptome Is Disrupted in Cardiorespiratory Muscles During Pancreatic Cancer Cachexia

In recent time course studies in the orthotopic KPC model, we outlined the time frame of cachexia development, identifying Day 12 postinoculation (in our hands) as a time point that reflects the onset of cachexia, defined by concurrent loss of body mass and skeletal muscle mass, and which is accompanied by both limb and diaphragm muscle fibre atrophy [16, 17]. Although cardiac wasting is not a diagnostic criterion for cachexia, it is increasingly recognized as a component of the syndrome in both patients and preclinical models [13, 15], including the orthotopic KPC model used herein [14, 17], where significant heart wasting is evident on Day 14, shortly after cachexia onset [17]. To determine whether cachexia is associated with circadian dysregulation in cardiorespiratory muscles, we collected diaphragm and heart muscles from Sham and KPC mice every 4 h over 24 h, beginning 12 days postinoculation and performed RNA‐seq. Consistent with our previous time course study in this model, tibialis anterior and gastrocnemius muscle masses were reduced in KPC mice relative to Sham at this timepoint, with tumour masses consistent with what we observed previously for the mild‐to‐moderate cachexia stage (Figure S1) [16, 17].

**FIGURE 1:**
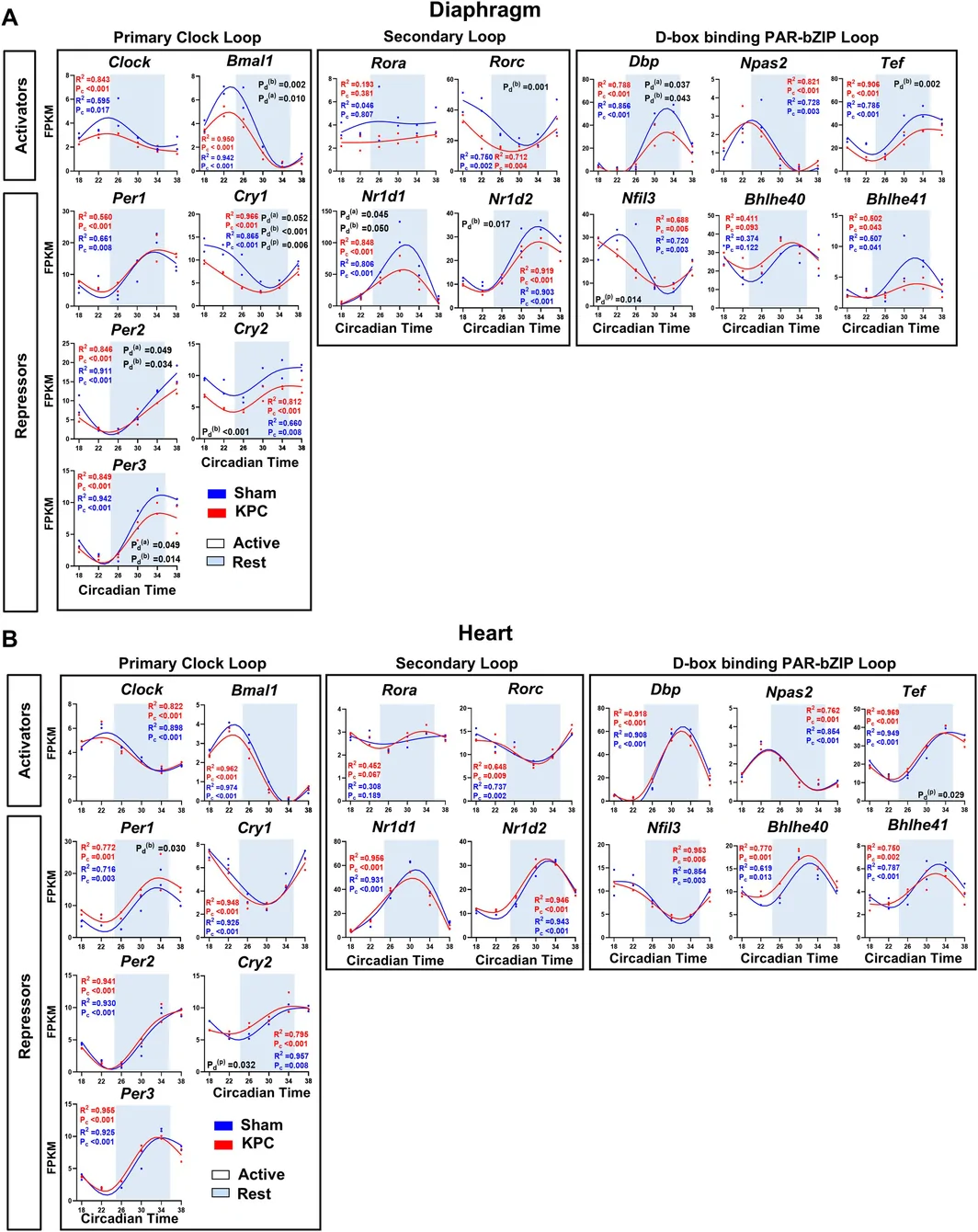
Core clock gene rhythmicity in cardiorespiratory muscles during pancreatic cancer cachexia. Circadian expression levels of core clock genes in the diaphragm (A) and heart (B) of Sham and KPC mice collected at a time point previously established to reflect cachexia onset were normalized by fragments per kilobase of exon per million mapped fragments (FPKM). Circadian rhythmicity (*P*_*c*_ < 0.01), differences in circadian patterns for amplitude (*P*_*d*_^(*a*)^ < 0.05), phase (*P*_*d*_^(*p*)^ < 0.05) and basal expression (*P*_*d*_^(*b*)^ < 0.05) were detected across circadian times (CT) 18, 22, 26, 30, 34 and 38 using R packages LR_Rhythmicity and LR_diff. Goodness of sinusoidal wave fitting (*R* ^2^). For nocturnal mice, CT18 corresponds to the middle of the active phase, while CT30 corresponds to the middle of the rest phase. The rest phase is denoted by a blue background. *N* = 2 mice/group/time point.

**FIGURE 2:**
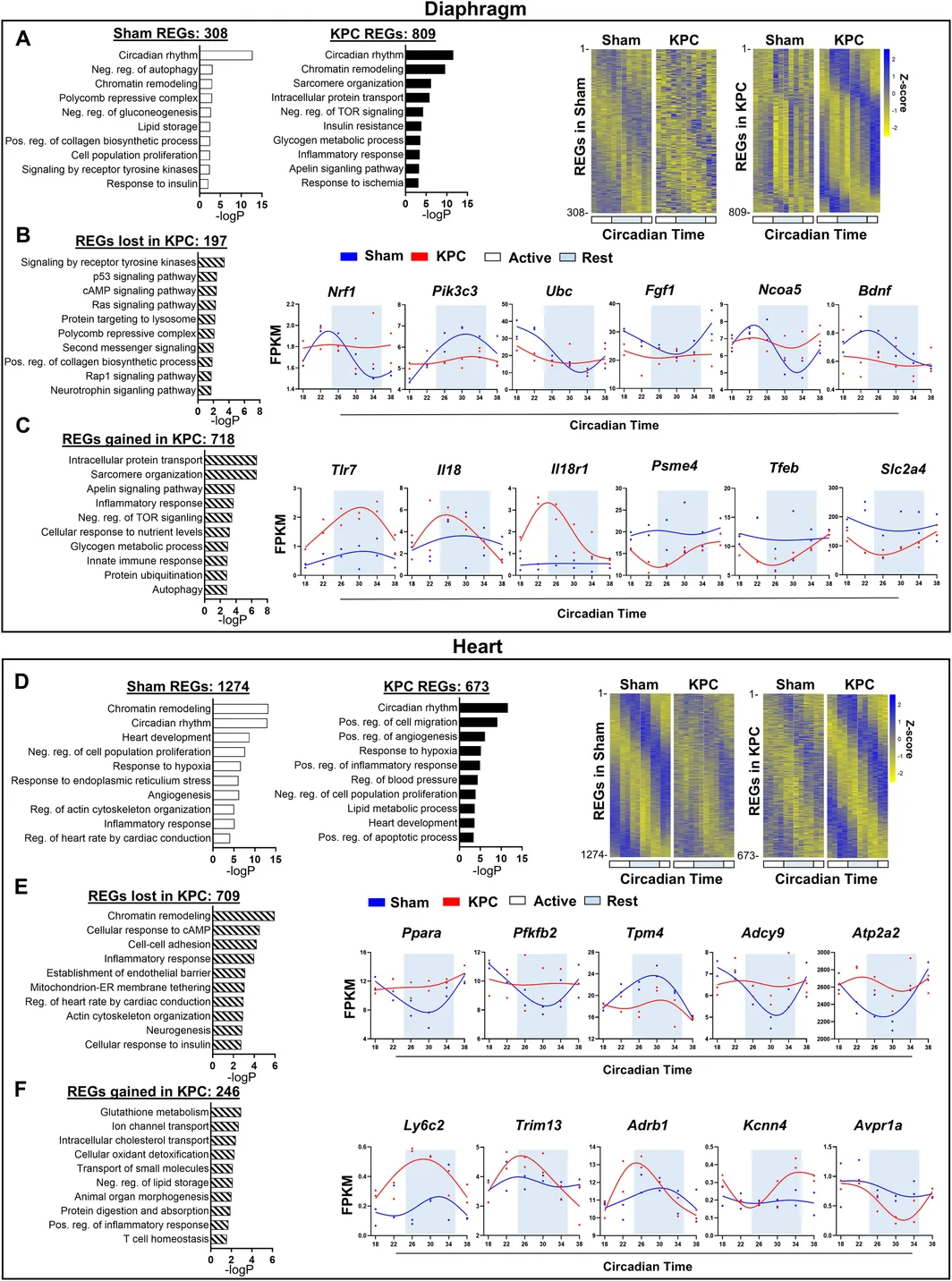
Tissue‐specific remodelling of circadian gene programs in cardiorespiratory muscles during pancreatic cancer cachexia. Top‐enriched Gene Ontology, KEGG and Reactome annotations based on REGs identified in the diaphragm (A) and heart (D) of Sham and KPC mice on D12, a timepoint previously established to correspond to cachexia onset, with corresponding heat maps of *z*‐scored REGs in each condition for both tissues. Light bars indicate active phase, while blue bars indicate rest phase. Biological processes of rhythmically expressed genes (REGs) that are lost (B, E), along with select examples, or gained (C, F), along with select examples, in the diaphragm and heart in response to pancreatic cancer. Circadian expression levels were normalized by fragments per kilobase of exon per million mapped fragments (FPKM) between conditions. Circadian rhythmicity (*P*_*c*_ < 0.01) was detected across circadian times (CT) 18, 22, 26, 30, 34 and 38 using R packages LR_Rhythmicity. Goodness of sinusoidal wave fitting (*R* ^2^). Genes that lost rhythmicity in KPC mice were defined as genes displaying a circadian rhythmicity statistic of *P*_*c*_ < 0.01 in Sham mice, and *P*_*c*_ > 0.10 in KPC mice. For nocturnal mice, CT18 corresponds to the middle of the active phase, while CT30 corresponds to the middle of the rest phase. The rest phase is denoted by a blue background. *N* = 2 mice/group/time point.

Diaphragm and heart RNA‐seq data were analysed using the LR_rhythmicity and LR_diff packages to identify REGs and differential circadian patterns in both tissues across conditions (Files S2 and S3). We first examined circadian expression of clock genes from the three interlocking circadian clock loops: primary, secondary, and D‐box binding PAR‐bZIP loops. As expected, core clock genes in the primary loop showed significant circadian rhythmicity in the diaphragm (Figure 1A) and heart (Figure 1B) of Sham mice. However, despite maintaining rhythmicity, many core clock genes were repressed in the diaphragm of KPC mice, exhibiting significant reductions in basal expression and/or amplitude compared to Sham mice. This included repression of *Bmal1*, a core clock gene essential for regulating rhythmic processes and maintaining muscle homeostasis [19]. In contrast, core clock gene rhythmicity in the heart was largely preserved, except *Per1*, which had elevated basal expression in KPC mice. We subsequently determined whether the circadian transcriptomes in the diaphragm and heart, which reflects the transcriptional output of the core clock mechanism with direct impacts on metabolism and physiology are also disrupted. In diaphragm muscles of Sham mice, 308 genes displayed significant circadian rhythmicity (i.e., REGs) (Figure 2A). Of these REGs, 64% (197/308) *lost* their circadian rhythmicity in KPC mice (Figure 2B). In contrast, we identified 718 genes that *gained* rhythmicity in diaphragm muscles of KPC mice, that did not display rhythmicity in Sham mice (Figure 2C).

To identify biological processes and pathways associated with genes that lost or gained circadian rhythmicity in the diaphragm of KPC mice we performed GO, KEGG, and Reactome enrichment analyses (Files S2 and S3). Genes losing circadian rhythmicity were enriched for processes related to cAMP signalling, lysosomal targeting, the Polycomb repressor complex and p53 signalling (Figure 2B). Circadian expression of REGs of interest that *lost* rhythmicity in diaphragms are shown in Figure 2B and include the transcription factor, *Nrf1*, which regulates mitochondrial homeostasis and cellular responses to proteotoxic, oxidative and ER stress; *Ncoa5*, a positive regulator of amino acid‐stimulated mTOR signalling; *Pik3c3*, a key mediator of autophagy and membrane trafficking; *Bdnf*, a myokine and master regulator of neuroplasticity; *Fgf1*, a positive modulator of glucose disposal and lipid metabolism; and *Ubc*, a stress‐inducible polyubiquitin precursor involved in removal of damaged/unfolded proteins. Analysis of the 718 genes that *gained* circadian rhythmicity within diaphragms of KPC mice revealed enrichment of processes related to chromatin remodelling and transcription, intracellular transport, sarcomere organization, inflammation, apoptosis, nutrient sensing and protein/organelle turnover, including autophagy and protein ubiquitination (Figure 2C). Circadian expression of genes of interest that *gained* rhythmicity in the diaphragm are shown in Figure 2C and include genes exhibiting time‐of‐day‐dependent increases in expression, such as *Tlr7*, which triggers innate immune responses to damage‐ and pathogen‐associated molecular patterns (DAMPs and PAMPs), and *Il18 and Il18r*, which participate in inflammatory signalling and cytokine secretion. In contrast, other genes gaining rhythmicity in the diaphragm displayed time‐of‐day‐dependent reductions in expression, including Glut4/*Slc2a4*, the major insulin‐regulated glucose transporter in muscle, and genes regulating proteostasis and cellular quality control, including *Psme4*, a stress‐responsive activator of the proteasome, and *Tfeb*, a master transcriptional regulator of the autophagy‐lysosome system.

In hearts of Sham mice, 1274 genes showed significant circadian rhythmicity (Figure 2D). Of these REGs, 56% (709/1274) *lost* their rhythmicity in KPC mice (Figure 2E). In contrast, hearts of KPC mice showed a *gain* in rhythmicity in 246 genes that did not display rhythmicity in Sham mice (Figure 2F). Notably, only 10% of REGs losing rhythmicity in hearts of KPC mice overlapped with those disrupted by cardiomyocyte‐specific *Bmal1* deletion [20], suggesting that factors extrinsic to the cardiac clock may contribute to circadian disruption in the heart. Enrichment analysis of the 709 REGs within the heart that *lost* circadian rhythmicity in KPC mice identified enrichment for processes related to chromatin remodelling and transcription, heart function (including beta‐adrenergic signalling and cAMP signalling), and cellular responses to insulin and neurogenesis (Figure 2E). Select REGs of interest that *lost* rhythmicity in hearts of KPC mice are shown in Figure 2E and include *Ppara*, a key regulator of cardiac fatty acid oxidation; *Pfkfb2*, a stress and nutrient‐sensitive regulator of glycolysis; *Adcy9*, a membrane‐bound enzyme involved in formation of cyclic AMP; and genes involved in regulating cardiac muscle contraction and relaxation, including tropomyosin 4 (*Tpm4*) and *Atp2a2*, which encodes the SERCA2 pump. Of the 246 genes that *gained* rhythmicity within the heart in response to KPC tumours, enriched processes were related to ion and cholesterol transport, and oxidant detoxification (Figure 2F). This included *Adrb1*, which encodes the β1‐adrenergic receptor, the predominant adrenergic receptor in cardiomyocytes responsible for mediating sympathetic responses including increased heart rate and contractility; and *Kcnn4*, which encodes a calcium‐activated potassium channel in cardiomyocytes that contributes to membrane repolarization and cardiac excitability. Though less apparent than in the diaphragm, several inflammatory transcripts also gained rhythmicity within the heart, exhibiting time‐of‐day‐dependent increases in expression, such as *Ly6c2*, a myeloid cell marker that plays a key role in immune cell activation and inflammatory responses and *Trim13*, an ER membrane anchored E3 ligase that mediates NF‐κB activation. In contrast, other genes gaining rhythmicity in response to KPC tumours displayed time‐of‐day‐dependent reductions in expression, including *Avpr1a*, which encodes the vasopressin receptor involved in regulating vascular tone and blood pressure.

### 3.2 Cachexia Is Associated With Time‐of‐Day‐Dependent Disruptions to Gene Networks That Support Cardiorespiratory Muscle Function

**FIGURE 3:**
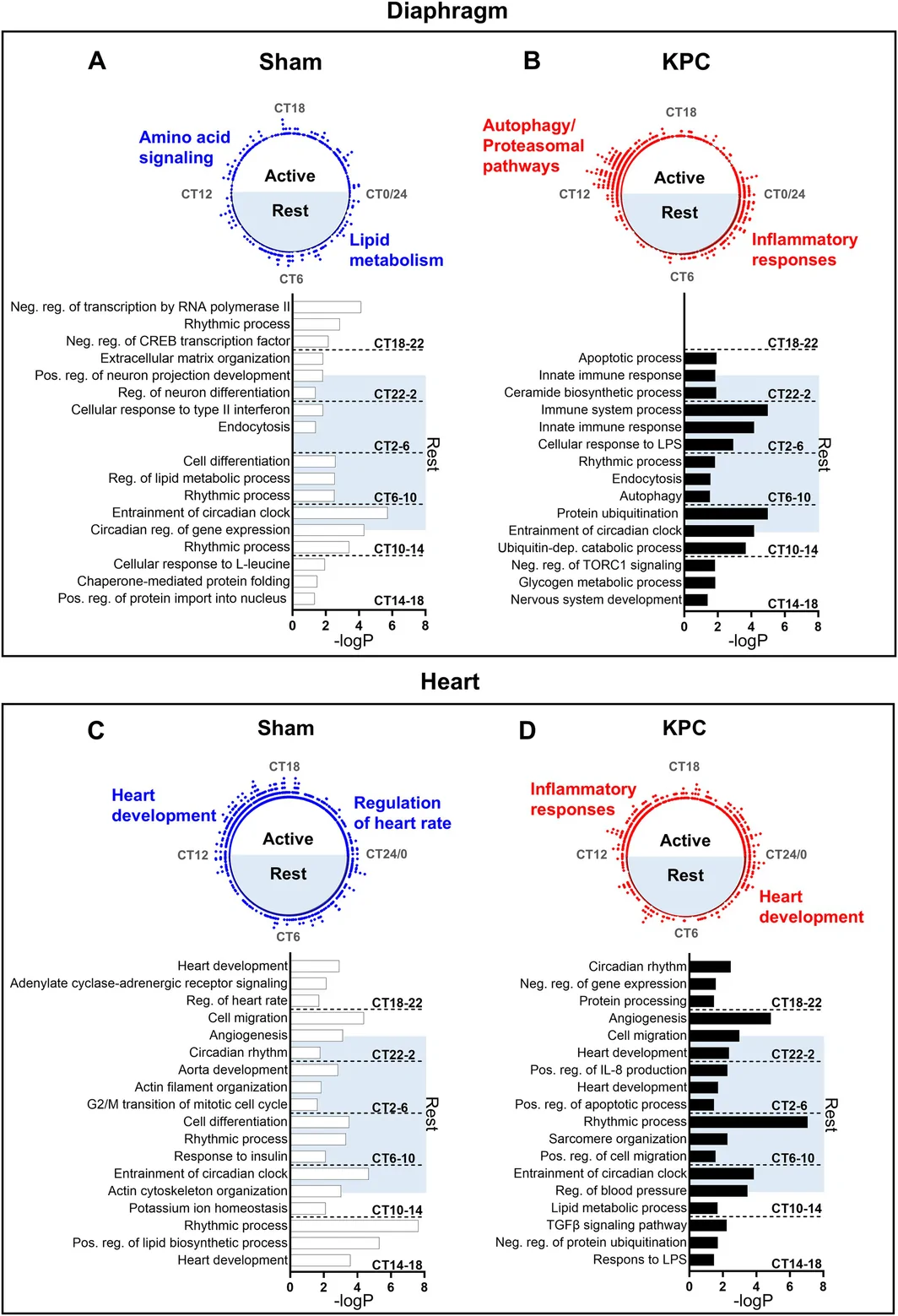
Temporal distribution of rhythmically expressed genes in cardiorespiratory muscles during pancreatic cancer cachexia. Peak time of expression maps of all rhythmically expressed genes (REGs) and their corresponding Gene Ontology biological processes in the diaphragm and hearts of Sham mice (A, C) and KPC mice during early‐stage cachexia (B, D). Each dot on the clock represents the time of peak expression for a single REG. Muscles were collected every 4 h across circadian times (CT) 18–38 for analysis in the LR_Rhythmicity package. For nocturnal mice, CT18 corresponds to the middle of the active phase, while CT30 corresponds to the middle of the rest phase. The rest phase is denoted by a blue background. *N* = 2 mice/group/time point.

Since we found disrupted circadian patterns of gene expression in both the diaphragm and heart, we next assessed the impact of this disruption on the timing and coordination of networks governing rhythmic biological processes in both tissues. Under normal homeostatic conditions, peak expression times of REGs are evenly distributed throughout the day, likely reflecting temporal distribution of biological functions. Our data in the diaphragm (Figure 3A) and heart (Figure 3C) of Sham mice support this. However, in diaphragms of KPC mice, the even distribution of peak REG expression is lost, with re‐clustering of REGs near activity offset (CT24, active‐to‐rest transition) and activity onset (CT12, rest‐to‐active transition) (Figure 3B). This re‐clustering of peak gene expression included a loss of rhythmic coordination of REGs governing key metabolic processes, such as *Regulation of lipid metabolism* (*Cnr1*, *Nr1d1*, *Nr1d2*) and *Cellular response to L‐leucine* (*Ep300*, *Ubr2*). Under normal conditions, pathways involved in lipid metabolism typically peak during the rest phase [6], whereas those involved in amino acid metabolism peak during the active phase [21]. However, in diaphragms of KPC mice, genes involved in these processes no longer peak in alignment with their natural rest/wake cycles. Instead, genes involved in inflammatory responses (*S100a8*, *Inava*, *Il18r1*) and ubiquitin‐dependent protein catabolism (*Ubr1/2/3*, *Ube3a*, *Trim54*) peaked at these times.

In the hearts of KPC mice, we found a limited effect on the temporal distribution of peak REG expression, which remained evenly distributed throughout the day. However, although the overall distribution of peak REG expression was unchanged, the timing of specific biological processes was altered. Under normal conditions, diurnal fluctuations are observed in heart rate, with higher heart rates and enhanced pacemaker activity during the active phase compared to the rest phase [22]. Our data in hearts of Sham mice support this, with genes involved in the *regulation of heart rate* (*Hcn4*, *Ednra*, *Pde4d*) peaking during the active phase. This temporal upregulation of genes involved in regulating heart rate was lost in hearts of KPC mice, with processes related to inflammatory responses (*Il33*, *Edn1*, *Hmgb2*) instead peaking during the active phase (Figure 3D).

**FIGURE 4:**
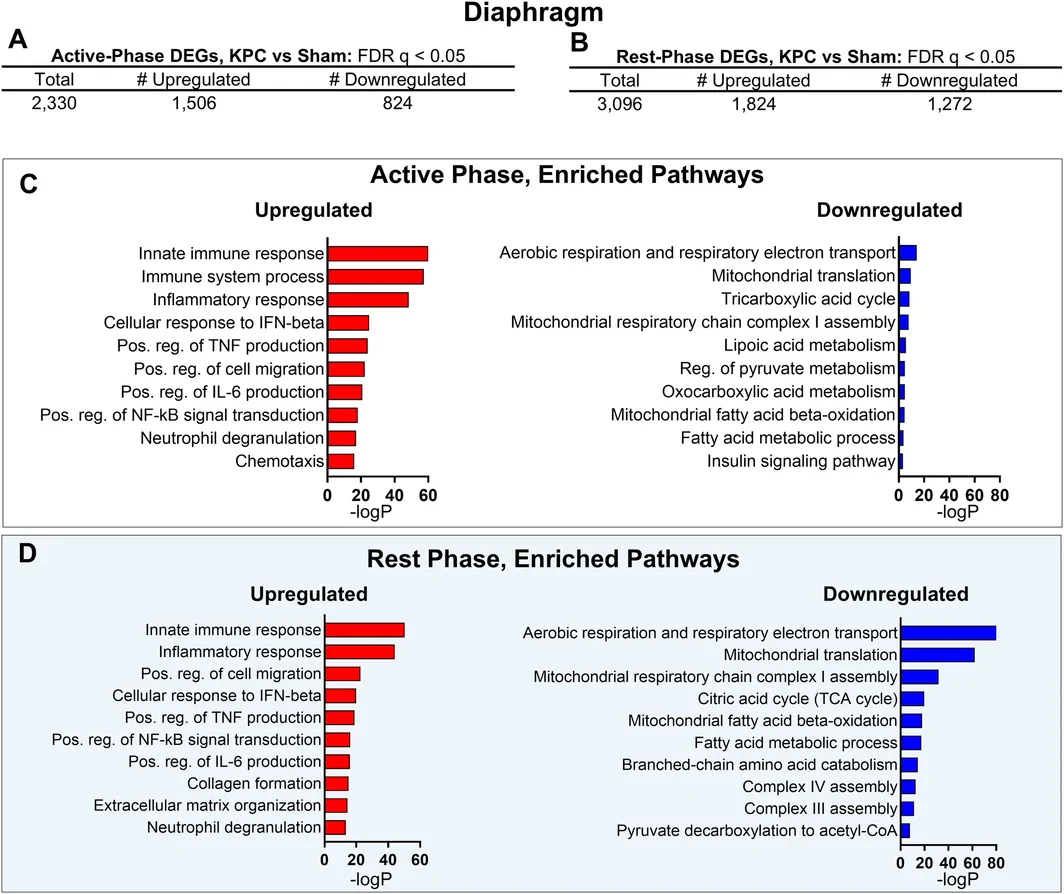
Time‐of‐day‐dependent effects on differentially expressed genes in the diaphragm during pancreatic cancer cachexia. Number of differentially expressed genes (DEGs) (*q* < 0.05) during the active phase (circadian time; CT18, CT22, CT38) (A) and rest phase (CT26, CT30, CT34) (B) in diaphragms of KPC vs. Sham mice. DEGs in each phase were analysed via DAVID to identify top‐enriched upregulated and downregulated GO biological processes, KEGG, and Reactome pathways (C,D). The rest phase is denoted by a blue background. *N* = 6 mice/group/phase.

**FIGURE 5:**
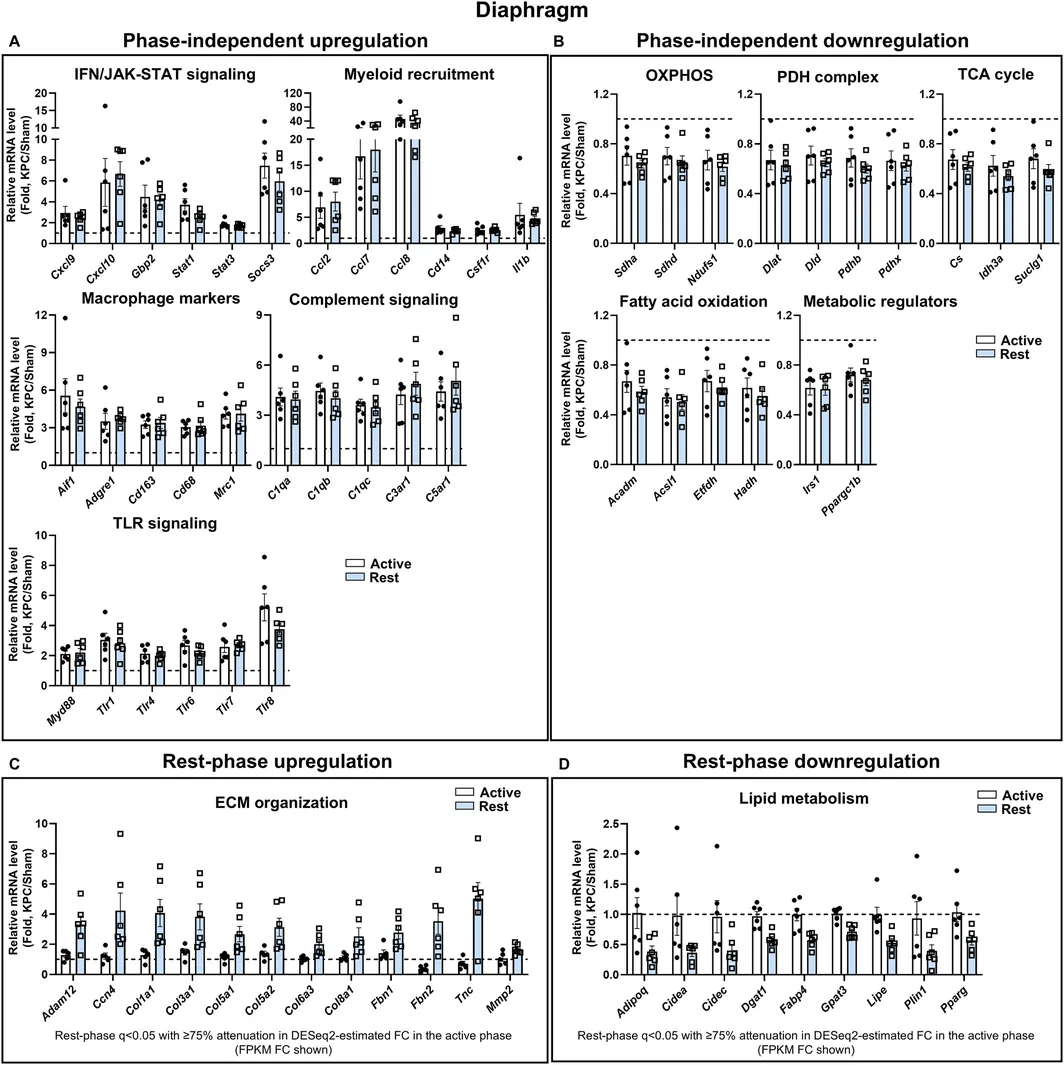
Phase‐independent and rest‐phase‐specific transcriptional responses in the diaphragm of KPC mice compared to Shams during pancreatic cancer cachexia. (A, B) Relative mRNA expression (FPKM) of representative phase‐independent upregulated genes and downregulated genes. (C, D) Relative mRNA expression (FPKM) of representative rest‐phase‐specific upregulated and downregulated genes. Phase‐specific genes were significantly differentially expressed in one phase (*q* < 0.05) with ≥ 75% attenuation of the DESeq2‐estimated fold change in the opposing phase. The dotted line reflects the Sham response, and the rest phase is denoted by the blue background. *N* = 6 mice/group/phase.

**FIGURE 6:**
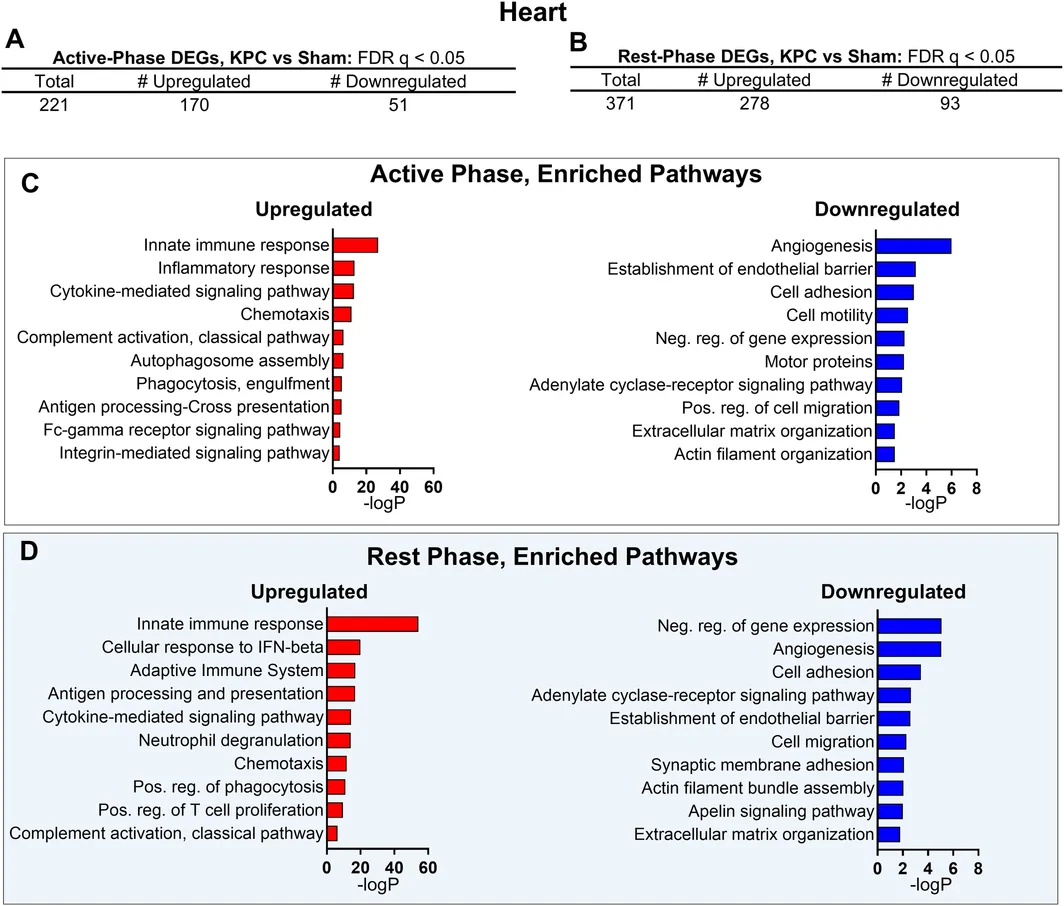
Time‐of‐day‐dependent effects on differentially expressed genes in the heart during pancreatic cancer cachexia. Number of differentially expressed genes (DEGs) (*q* < 0.05) during the active phase (circadian time; CT18, CT22, CT38) (A) and rest phase (CT26, CT30, CT34) (B) in hearts of KPC vs. Sham mice. DEGs in each phase were analysed via DAVID to identify top‐enriched upregulated and downregulated GO biological processes, KEGG, and Reactome pathways (C, D). For nocturnal mice, CT18 corresponds to the middle of the active phase, while CT30 corresponds to the middle of the rest phase. The rest phase is denoted by a blue background. *N* = 6 mice/group/phase.

**FIGURE 7:**
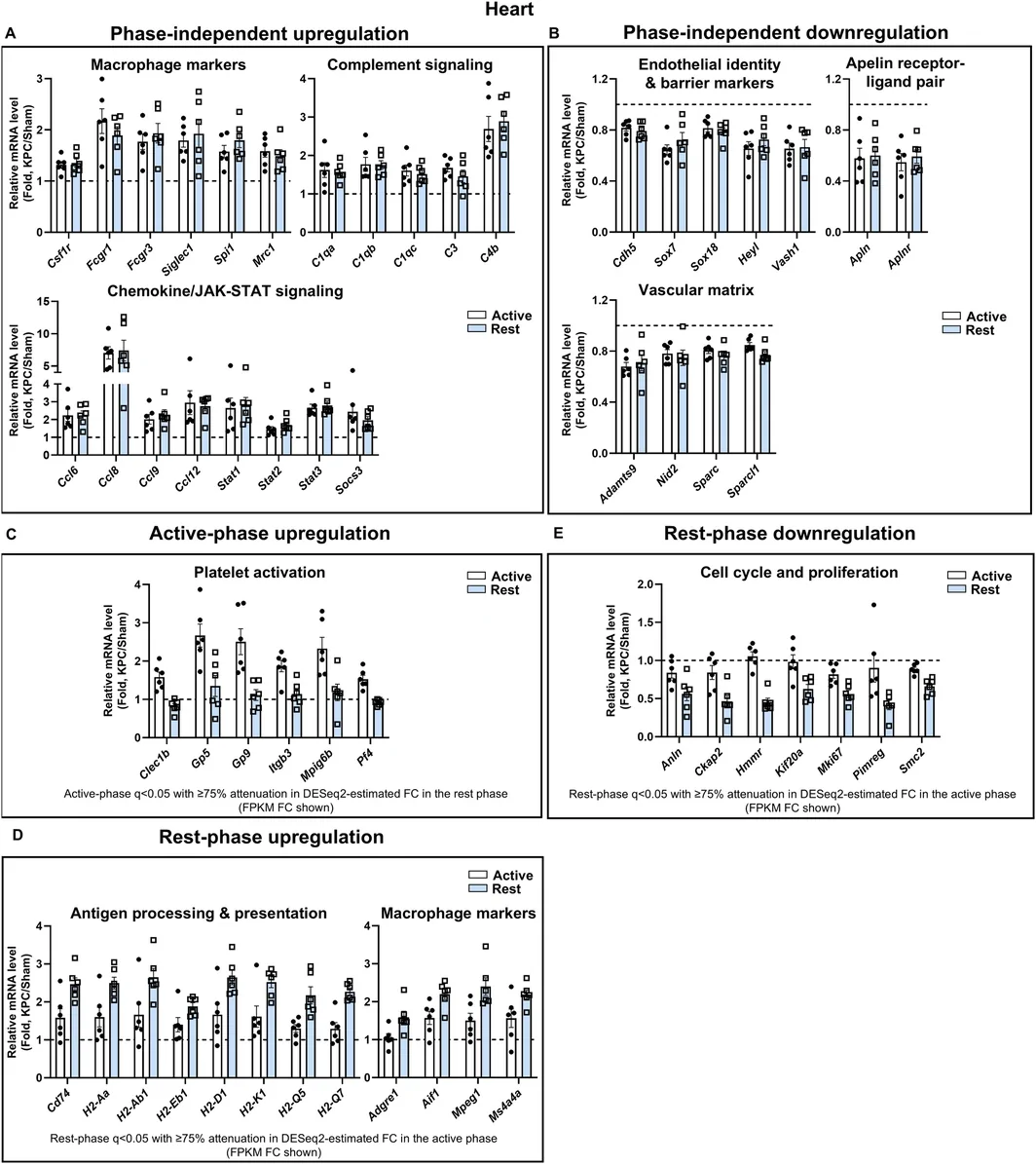
Phase‐independent and phase‐specific transcriptional responses in the heart of KPC mice compared to Shams during pancreatic cancer cachexia. (A, B) Relative mRNA expression (FPKM) of representative phase‐independent upregulated and downregulated genes. (C) Relative mRNA expression (FPKM) of representative active‐phase‐specific upregulated genes. (D, E) Relative mRNA expression (FPKM) of representative rest‐phase‐specific upregulated and downregulated genes. Phase‐specific genes were significantly differentially expressed in one phase (*q* < 0.05) with ≥ 75% attenuation of the DESeq2‐estimated fold change in the opposing phase. The dotted line reflects the Sham response, and the rest phase is denoted by the blue background. *N* = 6 mice/group/phase.

To further investigate time‐of‐day‐dependent transcriptional responses in the diaphragm and heart of KPC mice, we performed active and rest phase‐stratified transcriptomic analyses to identify DEGs (*q* < 0.05) between KPC and Sham mice within each phase (*N* = 6/group/phase, File S4). Consistent with our rhythmicity analyses, DEG responses showed time‐of‐day effects in the diaphragm (Figures 4 and 5), while similar but more limited effects were observed in the heart (Figures 6 and 7).

Phase‐stratified analysis of the diaphragm identified 2330 DEGs in the active phase (1506 upregulated genes, 824 downregulated genes) and 3096 DEGs in the rest phase (1824 upregulated genes, 1272 downregulated genes; Figure 4A,B). Both phases showed enrichment for innate immune activation and cytokine‐mediated programs among upregulated genes. While both phases showed enrichment of oxidative and mitochondrial programs of metabolism among downregulated genes (Figures 4C,D and S2), these processes were notably more enriched within the rest phase. Genes significant and concordant in direction across both phases were classified as phase‐independent. Phase‐independent upregulated genes were enriched for IFN/JAK–STAT signalling (*Stat1/3*, *Cxcl9/10*, *Gbp2*, *Socs3*), myeloid recruitment (*Ccl2/7/8*, *Cd14*, *Il1b*, *Csf1r*), macrophage markers and polarization programs (*Cd68*, *Aif1*, *Adgre1*, *Mrc1*, *Cd163*), complement signalling (*C1qa/b/c*, *C3ar1*, *C5ar1*) and toll‐like receptor signalling (*Tlr1/4/6/7/8*, *Myd88*; Figure 5A). Phase‐independent downregulated genes included those involved in oxidative phosphorylation (*Sdha/d*, *Ndufs1*), the pyruvate dehydrogenase complex (*Pdhb/x*, *Dlat*, *Dld*), the TCA cycle (*Cs*, *Idh3a*, *Suclg1*), and fatty acid oxidation (*Hadh*, *Acadm*, *Acsl1*, *Etfdh*), alongside regulators of mitochondrial biogenesis (*Ppargc1b*) and insulin signalling (*Irs1*; Figure 5B). To further identify phase‐specific transcriptional responses, we extracted genes showing significant upregulation or downregulation in one phase (*q* < 0.05) with ≥ 75% attenuation in DESeq2‐estimated fold change in the opposing direction during the other phase (Figure 5C–E; File S4). This revealed 63 upregulated and 27 downregulated genes meeting phase‐specificity criteria in the active phase, with no coherent pathway enrichment. In contrast, rest‐phase‐specific responses were more extensive and biologically coherent, with 88 upregulated genes enriched for extracellular matrix organization, including collagen genes (*Col1a1*, *Col3a1*, *Col5a1/2*, *Col8a1*, *Col6a3*) and ECM remodelling genes (*Postn*, *Fbn1/2*, *Tnc*, *Ccn4*, *Adam12*, *Mmp2*; Figure 5D), suggesting preferential upregulation of fibrosis‐associated transcriptional programs during the rest phase. Rest‐phase‐specific downregulated genes (*n* = 135) were enriched for lipid metabolism and fatty acid oxidation programs (*Pparg*, *Fabp4*, *Adipoq*, *Dgat1*, *Plin1*, *Lipe*, *Cidea/c*, *Gpat3*; Figure 5E), processes that normally peak during the rest phase in skeletal muscle from control mice [6, 23].

In the heart, phase‐stratified transcriptomic analysis identified 221 DEGs in the active phase (170 upregulated and 51 downregulated genes) and 371 DEGs in the rest phase (278 upregulated genes and 93 downregulated genes) (Figure 6A,B). Both phases showed enrichment for innate immune activation, complement signalling and cytokine‐mediated programs among upregulated genes and angiogenesis, endothelial barrier, extracellular matrix and muscle structural programs among downregulated genes (Figures 6C–F and S3). Phase‐independent upregulated genes included macrophage markers (*Csf1r*, *Fcgr1/3*, *Siglec1*, *Spi1*, *Mrc1*), complement components (*C1qa/b/c*, *C3, C4b*) and chemokine and JAK–STAT signalling mediators (*Ccl6/8/9/12*, *Stat1/2/3*, *Socs3*; Figure 7A), supporting phase‐independent innate immune activation in the heart. Phase‐independent downregulated genes included endothelial identity and barrier markers (*Cdh5, Sox7/18*, *Heyl*, *Vash1*), the cardioprotective apelin receptor‐ligand pair *Apln*/*Aplnr* [24] and vascular matrix components (*Adamts9*, *Nid2*, *Sparc* and *Sparcl1*; Figure 7B), suggesting altered endothelial homeostasis related to barrier function regardless of phase. Phase‐preferential responses in the heart included 45 upregulated and 15 downregulated genes in the active phase and 123 upregulated and 54 downregulated genes in the rest phase meeting the ≥ 75% attenuation criterion (File S4). Active‐phase‐specific upregulated genes were enriched for platelet activation (*Clec1b*, *Gp5*/*9*, *Itgb3*, *Mpig6b*, *Pf4*; Figure 7C), consistent with processes implicated in cancer‐associated thromboinflammation [25] and previous reports showing platelet activation peaks during the active phase [26]. Rest‐phase‐specific upregulated genes were enriched for MHC antigen processing and presentation (*Cd74*, *H2‐Aa*, *H2‐Ab1*, *H2‐Eb1*, *H2‐D1, H2‐K1*, *H2‐Q5, H2‐Q7*) and additional macrophage markers (*Adgre1*, *Aif1*, *Mpeg1*, *Ms4a4a*; Figure 7D), consistent with previously reported rest‐phase enrichment of immune cell antigen processing [27], thereby supporting increased engagement of macrophage‐associated transcriptional programs during the rest phase. Rest‐phase‐specific downregulated genes were enriched for cell‐cycle and proliferative programs (*Anln*, *Ckap2*, *Hmmr*, *Kif20a*, *Mki67*, *Pimreg*, *Smc2*; Figure 7E).

### 3.3 Repression of Core Clock Genes Occurs Prior to Cancer Cachexia Onset

**FIGURE 8:**
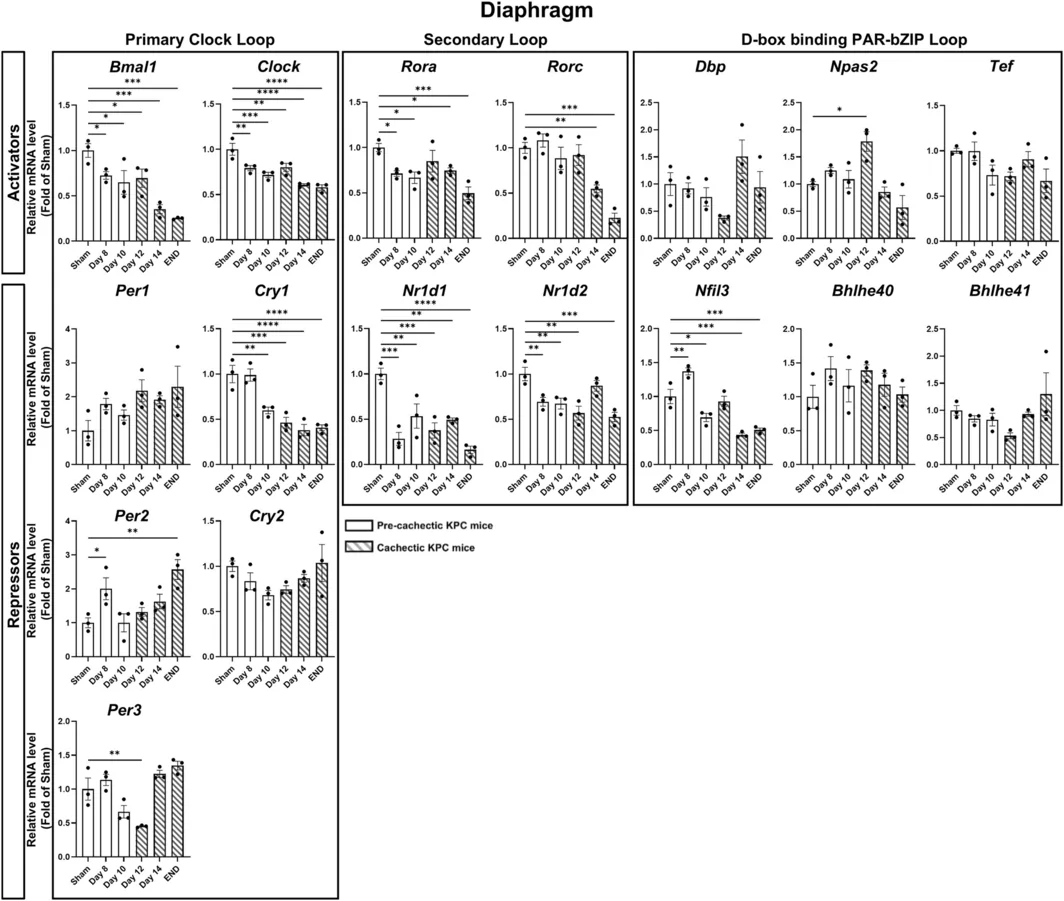
Repression of core clock activators in the diaphragm occurs prior to cachexia onset. Expression levels of clock genes in the diaphragm of KPC mice at various stages throughout the cachexia trajectory, including those reflective of pre‐cachexia (Days 8–10 post‐KPC cell inoculation), mild‐to‐moderate cachexia (Days 12–14) and at endpoint when cachexia is severe. Data were extracted from a previously published and fully characterized RNA‐seq time course dataset from diaphragm muscles of Sham mice and mice with orthotopic KPC tumours (GEO: GSE271521; Neyroud et al. [17]). Cachexia onset (dashed bars) is defined based on criteria established in the original study, which reported concurrent loss of body mass and peripheral skeletal muscle mass first occurring at Day 12 postinoculation. Complete phenotypic characterization of this cohort is shown in Neyroud et al. [17]. *N* = 3 mice/group/time point. *p < 0.05, **p < 0.01, ***p < 0.001, ****p < 0.0001.

To this point, our data demonstrate that the rhythmicity and expression level of clock genes are disrupted in the diaphragm at Day 12 postinoculation, at a time point previously shown to correspond to cachexia onset in the KPC model [16, 17], which may be involved in disruptions to circadian programs in this tissue. To assess clock gene expression disruption throughout the cachexia continuum, we extracted clock genes from our published RNA‐seq time course dataset from KPC mouse diaphragms collected at stages reflective of pre‐cachexia (Days 8–10 postinoculation), mild‐to‐moderate cachexia (Days 12–14) and at endpoint when cachexia is severe [17]. We found significant disruption in mRNA levels of most clock genes within the primary, secondary and D‐box binding PAR‐bZIP loops of the circadian clock system throughout the cachexia continuum (Figure 8). Notably, *Bmal1* and *Clock* were significantly repressed by Day 8 and remained suppressed thereafter, demonstrating transcriptional repression of essential circadian clock activators at stages prior to cachexia initiation and throughout later stages of cachexia. We also observed a marked repression of *Rora* and *Nr1d1* (which encodes REV‐ERB*a*) prior to cachexia induction, which are components of the secondary loop of the circadian clock system whose protein products have opposing effects on *Bmal1* transcription and help to stabilize the clock. Notably, both RORα [28, 29] and REV‐ERBα [30, 31] also have several clock‐independent functions and are critical for skeletal muscle metabolic health and mass maintenance. Taken together, these findings highlight early disruptions to clock components within the diaphragm during the pre‐cachexia phase that may contribute to disrupted circadian programming and alterations to muscle health that drive diaphragm wasting and remodelling.

## 4 Discussion

The purpose of this study was to examine changes in circadian patterns of gene expression within cardiorespiratory muscles during pancreatic cancer cachexia [16, 17]. The circadian clock mechanism exists in virtually all cells and consists of core clock genes that confer ~24‐h rhythms through transcription–translation feedback loops. These rhythms drive the cyclic expression of clock output genes to coordinate daily cellular activities and tissue‐specific functions. Through the studies performed herein, we demonstrate significant and unique disruptions to circadian patterns of gene expression in cardiorespiratory muscles that may contribute to wasting and dysfunction of these vital organs. These include time‐of‐day‐dependent repression of gene networks involved in oxidative metabolism, adrenergic signalling and muscle contractility, which were accompanied by an upregulation of inflammatory gene programs. We further found that the diaphragm exhibited significant repression and disruption to the rhythmicity of core clock genes, whereas core clock gene expression was largely preserved in the heart. Notably, we show that repression of key clock genes in the diaphragm occurs prior to cachexia development [17], supporting early circadian disruptions as a potential contributor to diaphragm wasting and dysfunction. Since both the diaphragm and heart are composed of multiple cell types and are influenced by systemic inputs including the autonomic nervous system (ANS), hormonal systems, brainstem respiratory centres and the central circadian clock [22, 32], rhythmic gene expression in these tissues likely reflects not only rhythms set by the intrinsic muscle clock and clocks of resident nonmuscle cells but also systemic cues that display circadian rhythmicity. Accordingly, the tissue‐specific variations in circadian gene expression observed within cardiorespiratory muscles may reflect alterations to both systemic inputs and peripheral clocks, acting either independently or through cross‐talk.

In general, the circadian clock is known to drive cyclic transcriptional programs that govern time‐of‐day‐dependent biological processes that help align with feeding and activity patterns, including those regulating amino acid, glucose and lipid metabolism in skeletal muscle [6, 21]. Consistent with this, across the diaphragm and *tibialis anterior* [12], we observed a shared disruption of circadian programs regulating oxidative metabolism, despite distinct metabolic demands of these muscles. Indeed, the diaphragm contracts continuously, whereas the *tibialis anterior* is intermittently active during locomotion, indicating that the disruption to oxidative programs of metabolism in tumour‐bearing mice is unlikely to be driven by reductions in muscle use. Specifically, across these skeletal muscles, genes involved in lipid metabolism and oxidative phosphorylation were substantially downregulated in the rest phase, a time when the expression of these genes normally peak [6]. This disruption could interfere with oxidative capacity potentially contributing to metabolic inflexibility and predisposing these muscles to fatigue, metabolic dysfunction and accumulation of toxic lipid intermediates (i.e., lipotoxicity) [33]. Disruptions to circadian programs influencing the capacity for lipid oxidation may be particularly detrimental to skeletal muscle during cancer, as a shift towards lipids as the primary fuel source for oxidative metabolism has been documented in several mouse models of cancer cachexia [34, 35]. Reduced capacity for lipid oxidation could potentially exacerbate any lipotoxic effects related to lipid oversupply, such as mitochondrial dysfunction and ROS production, activation of cellular stress pathways and induction of inflammatory signalling [33]. Notably, in each skeletal muscle, transcriptional repression of oxidative metabolic programs coincided with peaks in inflammatory programs during the rest phase, supporting potential cross‐talk between these events.

In the heart of KPC mice, we observed a loss of rhythmicity in genes governing adrenergic signalling and contractility, resulting in their downregulation during the active phase. Under normal physiological conditions, the rhythmic expression of these pathways helps prepare the heart for fluctuations in physiological demand by supporting heart rate, cardiac output and blood flow [22]. Indeed, cardiac function exhibits diurnal variation, with heart rate and cardiac output peaking during the active phase under elevated sympathetic tone and declining during rest with parasympathetic dominance [22]. Reduced left ventricular contractile and relaxation capacity has also been reported in cachectic KPC mice [13]. Together, these findings suggest that disruption of transcriptional networks supporting cardiorespiratory activity may be an underlying mechanism contributing to fatigue and impaired cardiac function in pancreatic cancer cachexia [13, 15]. The repression of gene networks involved in adrenergic signal transduction in the heart could reflect an adaptive response to desensitise the heart to chronic sympathetic outflow in tumour‐bearing hosts, including elevated circulating catecholamines [36], which promote lipid mobilization and have direct effects on cardiac metabolism and function [37]. Indeed, while sympathetic stimulation is acutely beneficial, sustained activation can promote maladaptive responses that contribute to oxidative injury and cardiac dysfunction. Alternatively, hyperactivity of the vagal nerve—which influences heart rate and contractility—could also be involved in the depression of circadian networks supporting cardiac function in tumour‐bearing hosts. In this regard, vagal nerve hyperactivity was recently identified as a mediator of cachexia in two different models of cancer cachexia [38], including the KPC model used herein, suggesting a potential link to the suppressed transcriptional programs governing cardiac function identified in the present study.

Among the muscles examined, the diaphragm exhibits the most severe disruption of its circadian clock. Under the same tumour burden, our previous findings in the *tibialis anterior* [12] and current heart data show altered clock output but largely preserved expression and rhythmicity of core clock genes. In contrast, the diaphragm shows pronounced repression and alterations to the rhythmic patterns of core clock components, including *Bmal1*, a key regulator of rhythmic biological processes [19]. The consequences of such disruption are well‐established from studies involving *Bmal1* deletion, which is sufficient to induce metabolic reprogramming, weakness and phenotypic changes in muscle associated with cachexia, including a metabolic switch away from carbohydrate metabolism, ultrastructural disorganisation of thick and thin filaments, insulin resistance, decreased mitochondrial volume and respiratory function [19]. We also observed repression of *Rora* and *Nr1d1* (encoding REV‐ERBα) prior to cachexia onset, mirroring the downregulation of *Bmal1*. These secondary loop components have opposing effects on *Bmal1* transcription that help stabilise clock oscillations. Critically, both REV‐ERBα and RORα also have well‐established clock‐independent roles in skeletal muscle that are directly relevant to the cachexia phenotype. In this regard, gain and loss of function studies have established REV‐ERBα as a positive regulator of mitochondrial biogenesis and oxidative metabolism in muscle that also represses atrophy‐associated genes and is critical for skeletal muscle mass maintenance [30, 31]. RORα has been shown to regulate lipid homeostasis in skeletal muscle [28] and its activation protects against pathological intramuscular fat accumulation by enhancing mitochondrial biogenesis and oxidative capacity [29]. Collectively, these findings thus support early repression of clock components in the diaphragm during the pre‐cachexia phase as a potential contributor to the decline in muscle health that may lead to diaphragm wasting, dysfunction and remodelling.

The basis for heightened disruption in the diaphragm remains unclear but may reflect its anatomical proximity to the pancreas, which could increase exposure to tumour‐derived factors that contribute to an enhanced inflammatory microenvironment and subsequent remodelling of muscle tissue architecture. In this regard, we previously found pronounced immune cell infiltration and upregulation of inflammatory programs in the diaphragm prior to cachexia initiation and throughout later stages of cachexia in the KPC model [17], consistent with our findings in rectus abdominis muscles of cachectic PDAC patients [7, 8, 39]. Inflammatory signalling through NF‐κB has been shown to suppress core clock gene expression and directly interfere with clock output through inhibiting CLOCK:BMAL1 activity [40]. Thus, it is possible that heightened inflammation in the diaphragm could contribute to its unique clock disruption. Furthermore, both REV‐ERBα and RORα have been shown to exert anti‐inflammatory functions through suppression of NF‐κB signalling [41, 42], suggesting that the early repression of these clock components within the diaphragm could further contribute to a pro‐inflammatory microenvironment prior to cachexia development. Although immune cell infiltration and activation of inflammatory programs are also evident in peripheral *tibialis anterior* muscles [8] and hearts [13] of cachectic tumour‐bearing mice, these phenotypes are notably less pronounced than what is observed in the mouse diaphragm, which is supported by our circadian RNA‐seq data collected from these tissues. While these findings could reflect distinct cachexia‐associated pathologies that develop in these muscles in response to cancer, they may also reflect differences in the timing in which pathology develops. In this regard, the loss of heart mass was not significant until after cachexia onset in this model, suggesting a slightly slower trajectory for cardiac muscle wasting [17]. Whether the comparatively limited disruption observed in the heart at this time point reflects a genuinely distinct pathological response or simply an earlier stage along a shared trajectory remains unclear. Phenotypic analyses of the heart across the full cachexia continuum, including measures of cardiomyocyte size and cardiac inflammation and remodelling, thus warrant further investigation, with the lack of such information representing a significant limitation of the current study. Moreover, due to the rapid freezing requirements of tissues to maintain circadian conditions and our use of the entire heart and diaphragm for circadian transcriptomics, phenotypic assessment of the diaphragm and heart from the animals used in the current study were not performed, which is also a significant limitation.

In summary, our findings establish a link between pancreatic cancer cachexia and circadian clock dysfunction in the diaphragm and heart, including time‐of‐day‐dependent alterations in gene networks regulating lipid and oxidative metabolism and proteostasis in the diaphragm and adrenergic signalling pathways in the heart. We further show that the disruption to these circadian programs coincided with an upregulation and gain of rhythmicity in inflammatory networks in both tissues, supporting inflammation as a potential mediator of circadian clock dysfunction in cardiorespiratory muscles that may contribute to wasting and dysfunction. We provide further evidence that core clock components are disrupted in the diaphragm of tumour‐bearing mice prior to cachexia development, supporting early disruptions to the core clock as a potential underlying mechanism contributing to circadian disruptions in this tissue. Importantly, the time‐of‐day‐dependent alterations in gene networks observed in cardiorespiratory muscles of pancreatic tumour‐bearing hosts support testing time‐of‐day‐based therapeutic interventions to maintain mass and function and further highlight the potential for therapeutic strategies that target circadian mechanisms. Consistent with this, recent work demonstrates that restoring hepatic circadian function in tumour‐bearing mice via REV‐ERBα/NR1D1 overexpression is sufficient to inhibit wasting of peripheral skeletal muscles and the heart [43], supporting a key role for circadian programs in mediating peripheral tissue cross‐talk and systemic homeostasis.

## Conflicts of Interest

The authors declare no conflicts of interest.

## Supporting information


**Figure S1:** Phenotypic characterization of animals in the circadian transcriptomic study. Initial body weight, tibialis anterior muscle mass, gastrocnemius muscle mass and tumour mass from Sham and KPC mice used in the circadian experiment. Both muscle masses were significantly reduced in KPC mice relative to Sham, supporting the presence of cachexia at the time of tissue collection. Tumour mass is consistent with the mild‐to‐moderate cachexia stage defined in Neyroud et al. [17]. Depending on data normality (Shapiro–Wilk test), two‐tailed Student's *t*‐test or Mann–Whitney U test was used to assess differences. ****p < 0.0001. Data show individual responses and mean ± SE.

**Figure S2:** Time‐of‐day‐dependent expression of genes within enriched pathways in the diaphragm of KPC mice. Heat maps show *z*‐scored expression of differentially expressed genes associated with selected enriched pathways identified from active‐ and rest‐phase upregulated and downregulated gene sets in the diaphragm. *N* = 6 mice/group/phase. The rest phase is denoted by the blue background.

**Figure S3:** Time‐of‐day‐dependent expression of genes within enriched pathways in the heart of KPC mice. Heat maps show *z*‐scored expression of differentially expressed genes associated with selected enriched pathways identified from active‐ and rest‐phase upregulated and downregulated gene sets in the heart. *N* = 6 mice/group/phase. The rest phase is denoted by the blue background.

**File S1:** RNA‐seq FPKM counts for all genes analysed in the circadian experiment, related to rhythmicity analyses. Counts for diaphragm and heart samples are provided on separate tabs. Samples are labelled by condition, time of tissue harvest and replicate mouse (ds00_1: diaphragm, Sham, CT18, replicate 1; K20_02: heart, KPC, CT38, replicate 2). For nocturnal mice, CT18 corresponds to the middle of the active phase, while CT30 corresponds to the middle of the rest phase.

**File S2:** Rhythmically expressed genes, rhythmicity analyses and temporal distribution of rhythmically expressed genes in the diaphragm. Output of the R package LR_Rhythmicity for all genes in the FPKM RNA‐seq dataset (File S1). Rhythmically expressed genes (REGs) in the diaphragms of Sham (cancer‐free) mice and mice with KPC pancreatic cancer were defined as *p* < 0.01 and are highlighted. Genes with *p* > 0.10 were considered nonrhythmic. Their corresponding biological processes are shown on separate tabs for each condition. Comparisons of REGs in each condition, along with the output of the R package LR_diff for differential circadian analyses between conditions, are included on separate tabs. Temporal distribution of the peak expression times of REGs and their corresponding biological processes in 4‐h increments over a 24‐h period are also included on separate tabs. For nocturnal mice, CT18 corresponds to the middle of the active phase, while CT30 corresponds to the middle of the rest phase.

**File S3:** Rhythmically expressed genes, rhythmicity analyses and temporal distribution of rhythmically expressed genes in the heart. Output of the R package LR_Rhythmicity for all genes in the FPKM RNA‐seq dataset (File S1). Rhythmically expressed genes (REGs) in the hearts of Sham (cancer‐free) mice and mice with KPC pancreatic cancer were defined as *p* < 0.01 and are highlighted. Genes with *p* > 0.10 were considered nonrhythmic. Their corresponding biological processes are shown on separate tabs for each condition. Comparisons of REGs in each condition, along with the output of the R package LR_diff for differential circadian analyses between conditions, are included on separate tabs. Temporal distribution of the peak expression times of REGs and their corresponding biological processes in 4‐h increments over a 24‐h period are also included on separate tabs. For nocturnal mice, CT18 corresponds to the middle of the active phase, while CT30 corresponds to the middle of the rest phase.

**File S4:** Differentially expressed genes identified in phase‐stratified transcriptomic analyses of the diaphragm and heart. Differentially expressed genes (DEGs; *q* < 0.05) identified between Sham and KPC mice within active and rest phases in the diaphragm and heart using DESeq2 (*n* = 6/group/phase). File includes all DEGs, phase‐independent DEGs and phase‐specific DEGs defined as genes significantly differentially expressed in one phase with ≥ 75% attenuation of the DESeq2‐estimated fold change in the opposing phase.

**File S5:** Supplementary references.

## Data Availability

Raw sequencing data are publicly available as of the date of publication and have the following accession numbers: GEO:GSE308727 (diaphragm) and GEO:GSE337972 (heart).
