# Supplementary material for: Pancreatic Cancer Disrupts Circadian Patterns of Gene Expression in Cardiorespiratory Muscles
Source: J Cachexia Sarcopenia Muscle. 2026 Sep 23;17(5):e70383. doi: 10.1002/jcsm.70383 (PMC13598680; doi:10.1002/jcsm.70383)
Supplement: Supplementary file 1 [file JCSM-17-e70383-s007.docx]

**Supplemental References**

S1.	Ding H, Meng L, Liu AC, Gumz ML, Bryant AJ, Mcclung CA *et al.* Likelihood-based tests for detecting circadian rhythmicity and differential circadian patterns in transcriptomic applications. *Brief Bioinform* 2021;**22**:bbab224.

S2.	Wolff CA, Gutierrez-Monreal MA, Meng L, Zhang X, Douma LG, Costello HM *et al.* Defining the age-dependent and tissue-specific circadian transcriptome in male mice. *Cell Rep* 2023;**42**:111982.

S3.	Hughes ME, Abruzzi KC, Allada R, Anafi R, Arpat AB, Asher G *et al.* Guidelines for Genome-Scale Analysis of Biological Rhythms. *J Biol Rhythms* 2017;**32**:380–393.

S4. Zong W, Seney ML, Ketchesin KD, Gorczyca MT, Liu AC, Esser KA et al. Experimental design and power calculation in omics circadian rhythmicity detection using the cosinor model. doi:10.1002/sim.9803.
